# Recent Advances: The Imbalance of Cytokines in the Pathogenesis of Inflammatory Bowel Disease

**DOI:** 10.1155/2017/4810258

**Published:** 2017-03-21

**Authors:** Qingdong Guan, Jiguo Zhang

**Affiliations:** Institute of Pharmacology, Taishan Medical University, Tai'an, Shandong Province 271016, China

## Abstract

Cytokines play an important role in the immunopathogenesis of inflammatory bowel disease (IBD), including Crohn's disease and ulcerative colitis, where they drive and regulate multiple aspects of intestinal inflammation. The imbalance between proinflammatory and anti-inflammatory cytokines that occurs in IBD results in disease progression and tissue damage and limits the resolution of inflammation. Targeting cytokines have been novel strategies in the treatment of IBD. Recent studies show the beneficial effects of anticytokine treatments to IBD patients, and multiple novel cytokines are found to be involved in the pathogenesis of IBD. In this review, we will discuss the recent advances of novel biologics in clinics and clinical trials, and novel proinflammatory and anti-inflammatory cytokines found in IBD with focusing on IL-12 family and IL-1 family members as well as their relevance to the potential therapy of IBD.

## 1. Introduction

Inflammatory bowel disease (IBD) is a chronic inflammatory disease of the gastrointestinal tract, which clinically contains Crohn's disease (CD), ulcerative colitis (UC), and other conditions [1, 2]. The inflammation of the intestinal mucosa in IBD is characterized by episodes of abdominal pain, diarrhea, bloody stools, weight loss, and the influx of neutrophils, macrophages, and other immune cells that produce cytokines, proteolytic enzymes, and free radicals that result in inflammation and ulceration [1, 3].

IBD is a lifelong disease occurring early in life in both males and females. The incidence and prevalence of IBD markedly increased over the second half of the twentieth century, and since the beginning of the twenty-first century, IBD has been considered one of the most prevalent gastrointestinal diseases [4–7]. Estimates indicate that as of 2005, about 1.4 million Americans and several millions persons worldwide have been diagnosed with IBD. Roughly 30% are children and adults between 10 and 30 years of age [8]. The incidence of CD in North America has been estimated at between 3.1 and 14.6 per 100,000, with a prevalence of between 26.0 and 198.5 per 100,000 [1]. For UC, both incidence and prevalence are estimated at between 2.2 and 14.3 and 37.5 and 229 per 100,000, respectively [1].

Although the cause of IBD remains unknown, considerable progress has been made in the recent years to unravel the pathogenesis of this disease. Studies have provided evidence that the pathogenesis of IBD is associated with genetic susceptibility of the host, intestinal microbiota, other environmental factors, and immunological abnormalities [9–11]. The immunological dysregulation in IBD is characterized by epithelial damage (abnormal mucus production, defective repair); expansion of inflammation driven by intestinal flora and a large number of cells infiltrating into the lamina propria including T cells, B cells, macrophages, dendritic cells (DC), and neutrophils; and a failure of immune regulation to control the inflammatory response [2, 4, 12]. A large number of soluble mediators are actively secreted by the activated lamina propria cells in the local tissue, including proinflammatory cytokines (TNF, IFN-*γ*, IL-6, IL-12, IL-21, IL-23, IL-17, integrin, etc.) and anti-inflammatory cytokines (IL-10, TGF*β*, IL-35, etc.) [2, 11]. CD is usually designated as a Th1 and Th17 condition with elevated production of IL-12, IL-23, IFN-*γ*, and IL-17, whereas UC is usually characterized as a Th2 and Th9 condition with increased production of IL-13, IL-5, and IL-9 [2]. The roles of cytokines in initiating, mediating, perpetuating, and controlling intestinal inflammation and tissue injury have been intensely studied because they are the key players in the pathogenesis of IBD and they may be the potential therapeutic targets [11, 13]. Antibodies against TNF, IL-12/IL-23p40, IFN-*γ*, IL-6R, IL-11, IL-13, IL-17A, integrin, and recombinant IL-10 and IFN-*β* have been tested or applied in clinics to treat IBD patients [11]. This review will describe recent advances in biologics treatment or clinical trials for IBD patients and novel cytokines found in the pathogenesis of IBD with focusing on IL-12 family and IL-1 family members.

## 2. Recent Advances in Biologics Treatment and Clinical Trials in IBD

Monoclonal antibodies against TNF are the first biologics approved and widely used for the treatment of both CD and UC, including infliximab, adalimumab, and certolizumab pegol, which have demonstrated good clinical efficacy in their abilities to induce remission and maintain steroid-free remission [14, 15]. However, around 20% of patients do not respond to anti-TNF, and over 30% eventually lose response [16]. Moreover, these antibody treatments can increase the risk of infections and malignancies [16]. Therefore, other new biologics are currently being developed for both anti-TNF-naive and TNF-resistant IBD patients [16]. In 2014, monoclonal antibody against integrin *α*4*β*7, vedolizumab, was approved to treat adults with moderate-to-severe active CD or UC. Vedolizumab also benefits one-third of patients with IBD who failed to previous anti-TNF therapy in a phase III trial [17]. Vedolizumab is efficacious and safe in pediatric IBD patients too, with UC patients experiencing earlier and higher rates of remission than CD patients in a multicenter experience trial [18]. Mongersen, an oral SMAD7 antisense oligonucleotide, which targets SMAD7 to increase the activity of TGF*β*, induces significantly higher rates of remission and clinical response than placebo in patients with active CD in a phase II trial [19]. Tofacitinib is an oral inhibitor of JAK1, 2, and 3 to block signaling pathways of *γ* chain-containing cytokines, including IL-2, IL-4, IL-7, IL-9, IL-15, and IL-21 [16]. In a phase II trial, tofacitinib induces clinical responses and remission in patients with moderate-to-severe UC, not for CD patients [20]. Add-on therapy with tralokinumab targeting IL-13 does not significantly improve clinical response but induces a higher clinical remission rate than placebo in a phase IIa trial, suggesting that tralokinumab may benefit some patients with UC [21]. But not all these biological agents achieve clinical responses. Another monoclonal antibody against IL-13, anrukinzumab, does not induce clinical benefit for patients with active UC in a phase IIa trial [22]. Several other biologics are under the clinical evaluation, such as anti-IL-12/IL-23p40.

## 3. IL-12 Family

IL-12 family of cytokines contains four cytokines, IL-12, IL-23, IL-27, and IL-35. Each member is composed of a helical *α* subunit (p35, p19, and p28) and a *β*-subunit (p40 and EBI3) structurally similar to the extracellular domain of type 1 cytokine receptors [23, 24]. IL-12 is composed of p35 and p40, IL-23 is composed of p19 and p40, IL-27 is composed of p28 and EBI3, and IL-35 contains p35 and EBI3 [23, 24]. The binding of the IL-12 family of cytokines to their corresponding receptors will activate JAK/STAT signaling pathways, leading to transcription of target genes that mediate biological activities. The IL-12 family of cytokines has emerged as important regulators of host immunity [23, 24].

IL-12 is predominantly produced by DC, monocytes, and macrophages following recognition of pathogenic structures by toll-like receptors and other receptors [25, 26]. IL-12 induces the production of IFN-*γ*, favours the differentiation of Th1, and forms a link between innate and adaptive immunity [27]. IL-23 is also predominantly produced by activated dendritic and phagocytic cells [26, 28, 29]. IL-23 plays an important role in stabilizing/amplifying Th17 proliferation [30, 31].

Studies have demonstrated that IL-12 and IL-23 play important roles in the pathogenesis of CD. Multiple studies indicate that IL-12 is overproduced in the gastric mucosa, lamina propria mononuclear cells, and macrophages in CD, and macrophages isolated from the inflammatory lesions of patients with CD produce increased amounts of IL-12 ex vivo [32–35]. Recent studies have highlighted the roles of IL-23 in the pathogenesis of CD [4, 36–38]. The colonic level of IL-23 is increased in patients with CD [39]. The myeloid DC from the mesenteric lymph nodes of CD patients secretes high levels of IL-23 [40]. The expression of IL-23R is upregulated in the lamina propria isolated from CD [41]. And the upregulated expression of IL-23R is correlated with IFN-*γ* [41]. Recently, a genome-wide association study has identified numerous SNP in *IL-23R*, with high association for CD and UC [42, 43]. Of interest, the G149R, V362I and R381Q IL-23R*α* chain variants, confers the protective effects in patients with CD and UC [43, 44]. These protective effects are due to impaired protein stability and intracellular trafficking, then leading to decrease the surface receptor expression and further reduce STAT signaling pathway [44]. Yen et al. used IL-10 knockout mice, a spontaneous IBD model, and showed that the development of colitis was suppressed by IL-23p19 deficiency but not IL-12p35 deficiency in IL-10^−/−^ mice; administration of IL-23 accelerated the onset of colitis and promoted inflammation through IL-17- and IL-6-dependent mechanisms [36]. As IL-12 and IL-23 share p40 subunit, targeting IL-12/IL-23p40 has been widely tested to improve intestinal inflammation in preclinical studies and clinical trials. Briakinumab and ustekinumab are human monoclonal antibodies against IL-12/IL-23p40 which induced clinical response and remission in a certain subtype of patients with CD [45–48]. In a phase IIb trial, briakinumab induced numerically greater rates of remission and response in moderate-to-severe active CD patients when compared to placebo treatment [47]. In a phase III-randomized trial, ustekinumab treatment induced significant clinical response and remission in moderate-to-severe CD patients refractory to prior TNF antagonists [48]. Since CD is a chronic inflammatory disease, it usually needs long-term treatment while monoclonal antibodies have short half-life. To overcome these disadvantages, we developed an IL-12/IL-23p40 peptide-based vaccine which induced relative long-lasting antibodies against IL-12/IL-23, and immunization with this vaccine improved TNBS-induced acute and chronic murine colitis by downregulating IL-12, IL-23, TNF, and IFN-*γ* and reducing fibrosis [49, 50]. Interestingly, immunization with the same vaccine can also ameliorate allergic murine skin and airway inflammation [51].

IL-27 is predominately produced by DC, macrophages and monocytes following stimulation by different immune stimuli [52, 53]. IL-27 regulates both innate and adaptive immune responses, including activating innate immune cells (e.g., macrophages), promoting Th1 and type 1 regulatory T cell differentiation, and inhibiting the differentiation of Th2, Th17, and Treg cells [52, 53]. However, under certain conditions, the regulatory function of IL-27 can be deviated, such as inhibiting Th1 but promoting Treg development [52, 53].

The important role of IL-27 in the pathogenesis of IBD has been indicated. Multiple studies have implicated IL-27 gene polymorphism and mutation are associated with the risk of IBD [54]. IL-27 gene expression level is increased in the local colon tissue of patients with active UC or active CD [55]. The proinflammatory and anti-inflammatory effects of IL-27 have been observed in IBD [54]. Oral delivery of recombinant IL-27 food-grade bacterium *Lactococcus lactis* ameliorates the murine colitis in a T cell-dependent colitis model, including improving survival, decreasing clinical score and pathologic score, downregulating inflammatory cytokines, and increasing IL-10 [56]. Subcutaneous administration with IL-27 also attenuates TNBS-induced intestinal inflammation with reducing pathologic score, decreasing inflammatory cytokines, and inhibiting Th17 cells [57]. IL-27R^−/−^ mice exhibited earlier onset and significantly increased severity of intestinal inflammation when compared to wild-type controls after DSS treatment [58]. These studies demonstrated the protective role of IL-27 in IBD.

On the other hand, some studies indicated the proinflammatory roles of IL-27 in IBD based on blocking IL-27 receptor signaling pathway [54]. IL-27R*α*^−/−^ mice developed less severe intestinal inflammation after DSS treatment when compared to wild-type mice, characterized by reducing inflammatory cytokines (IL-6, TNF, and IFN-*γ*). Another study showed that IL-27R*α*^−/−^ effector T cells had poor proliferation and less IFN-*γ* secretion; transfer of IL-27R*α*^−/−^ T cells results in diminished weight loss and reduced intestinal inflammation when compared to transferring of wild-type T cells [59]. Visperas et al. showed that IL-27R*α*^−/−^TCR*β*^−/−^ recipients did not develop intestinal inflammation after naive CD4^+^T cell infusion because these recipient mice had poor Th17 differentiation and lower expression of IL-6 and IL-1*β* by antigen-presenting cells; while IL-27R*α*^+/+^TCR*β*^−/−^ recipients developed severe colitis after naive CD4^+^T cell infusion [60]. These results indicate the complicated roles of IL-27 in IBD, which requires further investigation.

*IL-35* is a newly identified cytokine of the IL-12 family, which is mainly produced by Treg, activated B cells, and DC [11, 23, 61]. IL-35 can induce naive human and mouse T cells to differentiate into regulatory T cells (iTr35 cells), which do not express Foxp3, IL-10, and TGF but suppress T cell responses through IL-35 [23]. IL-35 suppresses T cell proliferation by inducing cell cycle arrest in G1 phase without inducing apoptosis [23]. In addition, IL-35 promotes the differentiation of human B cells into Breg which produces IL-35 and IL-10, and IL-35-producing B cells play an important role in the suppressive regulation of immunity [62]. Multiple studies have demonstrated the important regulatory role of IL-35 in IBD. Recently, two groups showed that IL-35 levels are significantly decreased in the serum but increased in the local colon tissue, and high IL-35 secreting intestinal Breg and circulating regulatory CD4^+^T and CD8^+^T cells are found in patients with active IBD [63, 64]; the level of IL-35 in the serum is inversely correlated with that of the activity of UC [64]. In T cell-dependent murine colitis model, EBI3^−/−^ mice (lacking both IL-27 and IL-35) develop early onset and severe colitis with shorten survival time, when compared to IL-27p28^−/−^ mice (lacking IL-27 only) [65], while recombinant IL-35 treatment significantly limited the development of several forms of experimental colitis and decreased levels of markers of Th1 and Th17 cells [65]. Adoptive transfer of IL-35-induced iTr35 can ameliorate the development of murine colitis [23]. These results suggest IL-35 may be an important regulator of IBD, with the potential for the treatment of IBD.

## 4. IL-1 Family

IL-1 family includes seven agonistic cytokines (IL-1*α*, IL-1*β*, IL-18, IL-33, IL-36*α*, IL-36*β*, and IL-36*γ*), three receptor antagonists (IL-1R*α*, IL-36R*α*, and IL-38), and one anti-inflammatory cytokine (IL-37) [66]. The IL-1 receptor family members include 10 molecules, from IL-1R1 to IL-1R10 [66]. Upon cytokine binding, IL-1 receptors heterodimerize, which recruit intracellular signaling molecules, including MyD88, IRAK, and TRAF6, then activate NF-*κ*B, p38, JNK, and/or MAPK transcription factors, leading to transcription of target genes (such as IL-6, IL-5, IL-4, IL-8, MCP-1, and COX-2) [67]. All innate immune cells express and/or are affected by the IL-1 family members. Moreover, the IL-1 family members play an important role in the differentiation and function of polarized innate and adaptive lymphoid cells [66]. Here, we will focus on the review of the roles of novel cytokines IL-33, IL-36, and IL-37 in the pathogenesis of IBD.

IL-33 is widely expressed by many cell types, such as epithelial cells, fibroblasts, smooth muscle cells, and endothelial cells [66, 68]. IL-33 exerts its biological function through binding of its receptor T1/ST2 [69]. Many immune cells are responsive to IL-33 and express T1/ST2 on their surface, and the main effects of IL-33 are involve in inflammation and type 2 immunity, including activation and accumulation of type 2 innate lymphoid cells, Th2, and M2 polarized macrophages [66, 68]. It has shown that IL-33 plays a role in the pathogenesis of many diseases, such as infection and autoimmune diseases [69].

IL-33 is constitutively expressed in epithelial cells at barrier sites of the gut, which can be regulated by epidermal growth factor [70]. In response to tissue damage, IL-33 functions as an alarmin by driving Th2, Th17, and Treg responses and influencing wound healing of damaged tissue [68, 71]. Through screening over 1500 IBD patients, a recent study shows that IL-33 polymorphisms contribute to the risk of IBD [72]. IL-33, along with ST2, is significantly increased in the inflamed IBD biopsy samples, especially in UC [72–74]. Furthermore, the expression of IL-33 is correlated with the inflammatory status [69]. At the disease remission stage of UC after anti-TNF treatment, IL-33 loses its expression in colonic crypts [75]. Soluble ST2 is a sensitive marker of treatment response and clinical outcome of UC [76]. IL-33 is also found to be increased in the colon tissue of animal colitis models [77, 78]. Deficiency of ST2 protects mice from TNBS- and DSS-induced murine colitis [79]. Administration of recombinant IL-33 exaggerates the severity of DSS-induced acute colitis, which is associated with marked elevation of IL-4, IL-5, and IL-13, significant reduction of IL-17 and IFN-*γ* in the colon tissue, impairment of the epithelial barrier, and delay of wound healing of the injured colonic epithelia [79–81]. But another group shows that administration of IL-33 protects DSS-induced acute colitis through inducing group 2 innate lymphoid cells with the expression of IL-4 and IL-5 and growth factor amphiregulin [78]. In TNBS-induced acute colitis, IL-33 administration protects the intestinal inflammation by promoting Th2/Foxp3^+^Treg cells [77]. In DSS-induced chronic colitis, IL-33 ameliorates the intestinal inflammation through suppressing Th1 and Th17 responses [82]. However, in SAMP1/YitFc spontaneous chronic murine colitis model, IL-33 administration worsens the chronic intestinal inflammation by enhancing eosinophil infiltration and increasing pathogenic Th2 response [83]; these effects can be reversed by blockade of IL-33 signaling or depletion of eosinophils and required gut microbiomes [83, 84]. These animal findings indicate that IL-33 may confer protection from injury or lead to inflammation although the behind mechanisms remain largely undefined.

IL-36 contains three agonistic ligands (IL-36*α*, IL-36*β*, and IL-36*γ*) and one antagonist ligand (IL-36Ra) [85], which bind to the same heterodimeric receptor. Not like other members of the IL-1 family, IL-36 is mainly expressed in keratinocytes, bronchial epithelia, brain tissues, and monocytes/macrophages [85]. IL-17 and TNF can induce the expression of IL-36 in keratinocytes, and IL-22 can synergize these effects [86]. IL-36 receptor signaling activates DC and plays a role in polarizing helper T cell responses [85]. IL-36 induces the secretion of proinflammatory cytokines (IL-12, IL-6, IL-1*β*, and TNF) from bone marrow-derived DC, IL-4, IL-17, and IFN-*γ* from CD4^+^T cells [87]. IL-36*β* induces the secretion of IL-12 and IL-18 from human monocyte-derived DC, which subsequently leads to the proliferation of IFN-*γ*^+^T lymphocytes [88]. Studies have shown that IL-36 plays a role in the pathogenesis of asthma, autoimmune diseases, psoriasis, and other diseases [85].

The mRNA expression of IL-36*α* and IL-36*γ*, not IL-36*β*, is increased in the inflamed mucosa of IBD patients, especially in UC and in DSS-induced murine colitis [89, 90]. Further, it finds that T cells, monocytes, and plasma cells in inflamed mucosa of IBD patients are the source of IL-36*α* and IL-36*γ* [89]. IL-36*α* and IL-36*γ* induce the expression of CXC chemokines on human intestinal epithelial cell line HT-29 cells in dose-dependent and time-dependent manners [89]. IL-36R^−/−^ mice reduced the intestinal inflammation in DSS-induced acute colitis, associated with decreased innate inflammatory cell infiltration into the colon lamina propria [91]. Similarly, after infection with the enteropathogenic bacteria *Citrobacter rodentium*, IL-36R^−/−^ mice reduced innate inflammatory cell infiltration and increased bacterial colonization in the colon, with enhanced Th17 and reduced Th1 responses [91]. Another group shows that IL-36 signaling may be important in the resolution of intestinal damage [92]. After DSS treatment, IL-36R-deficient mice reduce intestinal inflammation but significantly delay the wound healing of colonic mucosa, which is associated with the reduction of neutrophil infiltration into the colonic mucosa and reduction of barrier-protective cytokine IL-22 in the colon [92]. Administration of an aryl hydrocarbon receptor agonist restores IL-22 expression and promotes full recovery from DSS treatment in IL-36R-deficient mice [84, 92]. These studies indicate that IL-36 signaling plays a role in the pathogenesis of IBD and post injury healing, with the potential to be the target for the treatment of IBD.

*IL-37* is expressed in diverse human tissues, such as skin, tonsil, placenta, breast, and melanoma [93–95]. IL-37 is induced in DC and peripheral blood mononuclear cells stimulated by TNF, IFN-*γ*, IL-1*β*, and several toll-like receptor agonists [96]. The binding of IL-37 to IL-18R*α*/IL-1R8 controls the regulators of cellular adhesion and migration such as FAK, Pyk2, and transcription factors such as NF-kB and MAPK to display the anti-inflammatory activities mediated via Smad3 and caspase-1 [93, 94]. Increased levels of IL-37 have been reported in multiple diseases by measuring IL-37 mRNA or protein in cells derived from patients or in serum, such as rheumatoid arthritis, melanoma, atopic dermatitis, and ankylosing spondylitis [94]. In some other diseases, IL-37 levels are found to be decreased, such as psoriasis, asthma, and allergic rhinitis [94].

There are several reports that evaluated the levels of IL-37 in IBD patients. The percentage of IL-37-secreting cells is higher in the inflamed intestine of active CD patients than that in active ulcerative and noninflamed control tissues [63]. Levels of IL-37 in the sera are increased in active IBD patients, which are conspicuously produced by circulating B cells, active natural killer cells, and monocytes [63, 64]. In pediatric IBD patients, IL-37 protein expression is increased in submucosal lymphoid cells and correlated with histological severity score of intestinal inflammation; another IL-1 family member, IL-18, shows the similar pattern as IL-37 in these patients [97]. In vitro experiment shows that IL-37b inhibits the TNF*α*-induced chemokine IP-10 expression in human colonic subepithelial myofibroblasts [98]. Animal studies demonstrate anti-inflammatory roles of IL-37 in colitis. Transgenic mice overexpressing human IL-37b (IL-37b-tg) exhibits reduction of DC activation and proinflammatory cytokine secretion after LPS stimulation. IL-37b-tg mice protect from DSS-induced colitis, characterized by decreasing clinical disease score, histological score, and TNF*α* and IL-1*β* production, but increasing IL-10 production in colon tissue [99]. If overexpressing IL-37b on mesenchymal stromal cells, it will greatly increase the therapeutic efficacy of mesenchymal stromal cells in DSS-induced murine colitis [100]. These results indicate that IL-37 may be useful for the treatment of IBD.

## 5. Conclusion and Prospective

Cytokines play a crucial role in driving, perpetuating, resolving, and wound healing of intestinal inflammation in IBD. Novel biologics that targeting cytokines or cytokine signaling pathway cascades are being used in clinics or being tested in clinical trials. However, these biologics only seem to have beneficial clinical effects in certain subgroups of IBD patients. This may reflect the complex of cytokine networks in the inflamed colon tissue, which are subject to types of inflammation, location, microbiota, genetic, immune cell plasticity, and others [11]. Blockade of a single cytokine in IBD patients may drive other proinflammatory cytokine pathways. Therefore, to optimize the clinical response and remission rate in IBD patients, it may require using multiple cytokine inhibitors that simultaneously block several cytokines or common cytokine signaling pathway—the JAK-STAT pathway. Taken together, anti-TNF and anti-integrin *α*4*β*7 have been the mainstay of biological therapy in IBD. New cytokine targets (e.g., IL-12/IL-23p40 and SMAD7), novel anti-inflammatory cytokines (e.g., IL-35 and IL-37), and personalized medicines may provide potential treatment for IBD patients.

## Abbreviations

CD: Crohn's disease
DC: Dendritic cells
DSS: Dextran sulfate sodium
IBD: Inflammatory bowel disease
IFN: Interferon
IL: Interleukin
TNBS: 2,4,6-Trinitrobenzenesulfonic acid
TNF: Tumor necrosis factor
UC: Ulcerative colitis.
